# On-Line Monitoring of Radiocarbon Emissions in a Nuclear Facility with Cavity Ring-Down Spectroscopy

**DOI:** 10.1021/acs.analchem.1c03814

**Published:** 2021-11-24

**Authors:** Johannes Lehmuskoski, Hannu Vasama, Jussi Hämäläinen, Jouni Hokkinen, Teemu Kärkelä, Katja Heiskanen, Matti Reinikainen, Satu Rautio, Miska Hirvelä, Guillaume Genoud

**Affiliations:** † VTT Technical Research Centre of Finland Ltd, P.O. Box 1000, FI-02044 Espoo, VTT, Finland; ‡ Fortum Power & Heat Oy Loviisan Voimalaitos, P.O. Box 23, 07901 Loviisa, Finland

## Abstract

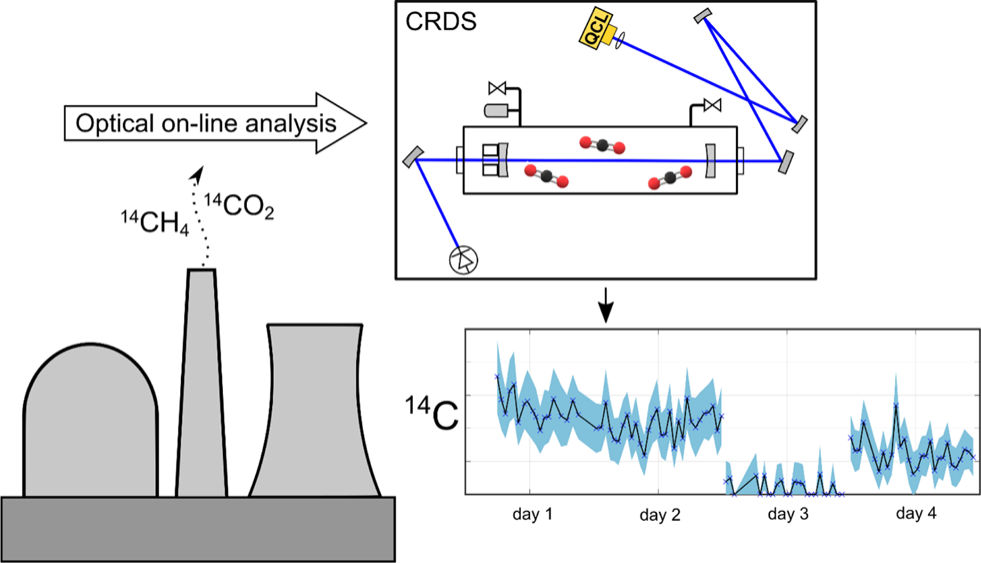

There are currently no suitable methods for sensitive automated in situ monitoring of gaseous radiocarbon, one of the main sources of radioactive gas emissions from nuclear power plants. Here, we present a transportable instrument for in situ airborne radiocarbon detection based on mid-infrared cavity ring-down spectroscopy and report its performance in a 1-week field measurement at the Loviisa nuclear power plant. Radiocarbon is detected by measuring an absorption line of the ^14^CO_2_ molecule. The time resolution of the measurements is 45 min, significantly less than the few days’ resolution of the currently used technique, while maintaining a comparable sensitivity. The method can also assess the prevalence of radiocarbon in different molecular species in the airborne emissions. The optical in situ monitoring presented is a completely new method for monitoring emissions from nuclear facilities.

## Introduction

Nuclear power plays an important role in mitigating climate change before renewable zero-emission energy sources are more widely used. The core technology is fundamentally free of greenhouse gas emissions and enables continuous, high-capacity energy production. Nuclear power plants (NPPs) are constantly monitored to ensure minimum impact on the environment, and most of the radionuclides arising from NPPs are already efficiently measured. However, emissions of gaseous beta-emitters, such as radiocarbon (C14) and tritium, are still challenging to monitor as a suitable method for their automatic on-line detection is lacking. In particular, radiocarbon emissions require monitoring because airborne radiocarbon can accumulate in photosynthesising organisms, such as plants used as human food.^1,2^ Due to its long half-life, C14 has a high residence time in the environment and requires long-term monitoring. Currently, radiocarbon monitoring is mostly based on liquid scintillation counting (LSC) or accelerator mass spectrometry (AMS).^3,4^ Although very sensitive, these techniques are essentially laboratory-based and cannot be reasonably converted to automated, field-deployable instruments. They both require prolonged sample collection, complex sample preparation, and labor-intensive analysis work, especially when analyzing gaseous samples. LSC is used in the nuclear industry, but the method suffers from overlapping scintillation peaks of other radionuclides, which therefore need to be separated chemically beforehand. Hence, there is a need for new technologies to ensure more efficient monitoring of radioactive gaseous emissions.

Radiocarbon is produced naturally at a constant rate in the upper parts of the atmosphere in the ^14^N(n,p)^14^C reaction by the interaction of atmospheric nitrogen with thermal neutrons produced by cosmic rays. At the same time, radiocarbon constantly decays with a half-life of 5700 years,^5^ resulting in a natural abundance of ^14^C/C = 1.2 parts per trillion (ppt). In a nuclear power plant, radiocarbon is produced in the same reaction from nitrogen by thermal neutrons from the reactor core as well as from ^17^O and ^13^C atoms via the ^17^O(n,α)^14^C and ^13^C(n,γ)^14^C reactions.^1,6,7^^14^N and ^17^O are present in reactor coolants, moderators, and fuels, while the ^13^C(n,γ)^14^C reaction occurs almost exclusively in graphite moderators. The abundance of thermal neutrons around the nuclear reactor core results in an increased mole fraction of radiocarbon, varying from below 1 parts per billion (ppb) to thousands of ppbs of ^14^C/C.^8,9^ In the gaseous form, the produced C14 is mostly bound to carbon dioxide and organic molecules such as methane. Their pathways in an NPP depend on the structure of the power plant, but typically, they are evacuated through the NPP stacks. The released C14 can be assimilated by living organisms outside the facilities, and therefore, C14 emissions must be monitored.^1,2^ The molecular form of radiocarbon determines how it affects the environment. In particular, CO_2_ is absorbed by all photosynthesizing organisms and is therefore a high risk for the environment, while CH_4_ is mainly a byproduct of organic activity and can be exploited only by specialized methanotrophic bacteria and archaea.^10^ During operation and decommissioning, NPPs also produce solid waste containing C14, which is stored in nuclear waste repositories. C14 is present in high concentrations in many types of waste, such as spent ion-exchange resins, reactor structures, and moderator graphite.^1,11^ Biodegradation of the waste material leads to gaseous C14 emissions, requiring in situ monitoring of the repositories.^1,12^

In many countries, monitoring of radiocarbon emissions from NPPs is required by nuclear safety regulations. However, none of the current detection methods can provide automated in situ monitoring nor can provide measurements with a good time resolution.^1,8,13^ In contrast to the conventional methods, laser absorption spectroscopy allows direct trace gas detection with high sensitivity and without interferences from other radionuclides. The use of optical methods offers several advantages in terms of size, cost, and usability over the current state of the art. In particular, high sensitivity can be achieved when using cavity-enhanced spectroscopy methods, such as cavity ring-down spectroscopy (CRDS).^14−16^ Excellent sensitivities for radiocarbon dioxide detection have been reported in laboratory measurements,^17−22^ and CRDS has been suggested for radiocarbon monitoring at nuclear facilities.^18,23−25^ However, in situ continuous radiocarbon measurements with these techniques have so far not been reported. In this work, we present the novel use of CRDS for continuous in situ monitoring of radiocarbon stack emissions from a nuclear power plant.

Besides radioactive emission monitoring, radiocarbon is of interest in other fields. Radiocarbon content is an indicator of the origin and age of a carbon-containing material, having completely decayed in fossil carbon, while biogenic carbon contains the natural abundance of 1.2 ppt. Therefore, radiocarbon is commonly used to date historical artifacts. Moreover, determining the radiocarbon content is an ideal solution for verification of biofraction in combusted materials that are mixed from multiple sources.^26,27^ Recently, the use of laser absorption spectroscopy was reported in quantifying biofraction in biofuels, where the method proved to be suitable for such applications.^28^ On-line radiocarbon monitoring at atmospheric concentrations with high temporal and spatial resolution can give invaluable information on the origin of atmospheric carbon dioxide. This allows for a better understanding of the contribution of carbon of the fossil origin to climate change and can be used to develop more advanced climate models. Eventually, atmospheric radiocarbon monitoring can be used as a tool for authorities to identify the producers of fossil emissions and enforce international climate agreements. Another significant application benefitting from the development of C14 detection is pharmacology, where C14 labeling of a drug molecule enables tracing its metabolic routes in the human body.^29^ In most of these applications, development of field-deployable instrumentation is essential.

## Methods

### Cavity Ring-Down Spectroscopy

Laser spectroscopy relies on detecting the light absorption of the species of interest at a specific wavelength. In CRDS, the absorption path length is increased by placing the gas sample in an optical cavity formed by two high-reflectivity mirrors, resulting in a high sensitivity. The light of a narrow-line-width laser is coupled between the two mirrors, resulting in light intensity buildup inside the cavity. After the light intensity reaches a set threshold, the light source is switched off, and the light in the cavity decays exponentially. In an empty cavity, the decay time, also known as the ring-down time, depends only on the light losses of the cavity. Additional losses due to the light absorption of a sample gas decreases the decay time. The wavenumber-dependent absorption coefficient, α(ν), can be calculated by comparing the vacuum ring-down time, τ_*0*_, with the ring-down time in the presence of the absorbing gas, τ(ν), as follows: α(ν) = 1/[*c*τ(ν)] – 1/[*c*τ_0_], where *c* is the speed of light and ν is the wavenumber. The measurement is independent of intensity fluctuations of the laser source as the exponential decay of light is fitted to determine the ring-down time.^14,16^ Another benefit of the technique is that it is self-calibrated as changes of τ relative to τ_0_ are detected to determine the mole fraction in the sample instead of measuring only the transmitted light intensity.

**Figure 1:**
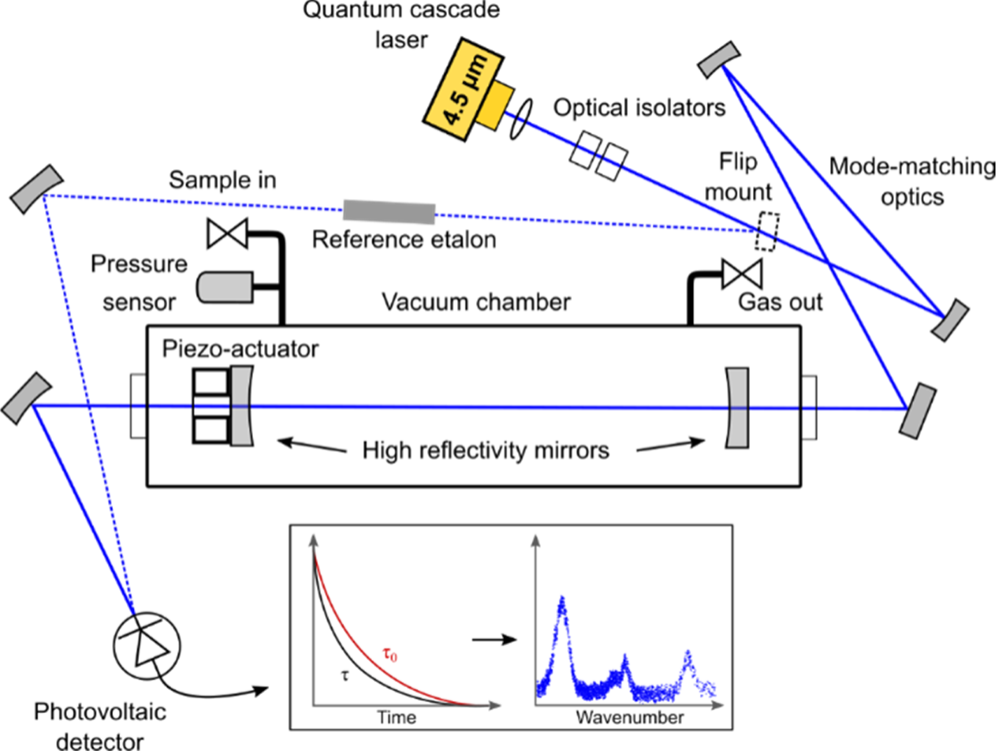
Schematic representation of the CRDS instrument for radiocarbon detection. The main path of the QCL laser is shown with the continuous blue line, while the dashed blue line represents the path for the laser wavelength calibration using a reference etalon.

A schematic of our CRDS instrument is shown in Figure 1. A narrow-line-width, single-frequency quantum cascade laser (QCL) L12004-2209H-C from Hamamatsu with a 4.527 μm central wavelength is used as a tuneable light source. Its lasing wavelength is tuned between 2208.6 and 2210.2 cm^–1^ by varying the laser driving current and the laser temperature. The laser beam is collimated by an aspheric lens and guided through two Faraday optical isolators of 30 dB isolation each to mode-matching optics and then to the cavity. The optical isolators minimize the optical feedback from the cavity back to the laser. Two isolators were used as the isolation from a single isolator was not sufficient. The laser TEM_00_ mode is matched to the cavity mode using two concave gold-coated mirrors. The cavity is formed by two high-reflectivity ZnSe mirrors with dielectric coating and a reflectivity of 99.97% and a radius of curvature of 1 m. The mirrors are situated 38 cm apart from each other. The second mirror is mounted on a tip/tilt platform controlled by piezo-electric actuators from Physik Instrumente. The mirrors and the piezo-controlled platform are enclosed inside a vacuum chamber to minimize variation of the mirrors’ positions in varying pressures. The 0.76 L chamber is sealed by two antireflective-coated CaF_2_ windows. The cavity is insulated with polyurethane foam, and its temperature is actively stabilized with a temperature controller regulating four Peltier elements. After the cavity, a spherical mirror focuses the light onto a HgCdTe photovoltaic detector from VIGO. The detector signal is recorded and digitized with a 250 MHz 14-bit FPGA card from National Instruments. The FPGA acquisition card also sends a trigger signal to the laser driver, when light intensity in the cavity reaches a set threshold level. This rapidly offsets the QCL to another wavelength and thus stops the light coupling into the cavity, which in turn initiates the light-intensity decay, that is, the ring-down event. The offset step was experimentally adjusted to minimize the exponential fit residual and the rate of out-filtered ring-down events. The ring-down events recorded by the FPGA card are automatically processed and fitted with an exponential function to extract the ring-down time using LabVIEW-based software. The acquisition software automatically filters out exponential fits with non-flat residuals resulting from higher-order cavity mode coupling and other noise sources. A scroll pump is used to evacuate the cavity, whose pressure is monitored with a capacitance manometer. The measured vacuum ring-down time is 3.95 μs corresponding to a cavity finesse of 9830. To record a spectrum, the QCL wavelength is scanned over the wavelength range of interest by ramping the laser driving current with a sawtooth waveform at a frequency of 40 Hz, while the QCL temperature is kept constant. A germanium etalon is used to calibrate the non-linear relationship between driving current and laser wavelength. The high finesse optical cavity, acting as a Fabry–Pérot interferometer, transmits light only at discrete wavelengths separated by its free spectral range. It is thus necessary to slowly scan the cavity length with the mirror on the piezo-controlled platform to increase the wavelength resolution of the measurement. All the optics of the setup are fitted on a 45 × 60 cm Nexus optical board, which is positioned on the top level of a moveable 19-inch instrument rack with an overall size of 110 cm × 80.5 cm × 60.5 cm (height × depth × width). The electronics, data acquisition, power supplies, and pump are positioned in two levels beneath the optical board and cooled down by two fans flowing air through the rack. A computer-aided design of the rack assembly is presented in the Supporting Information.

### On-Line Sample Processing

**Figure 2:**
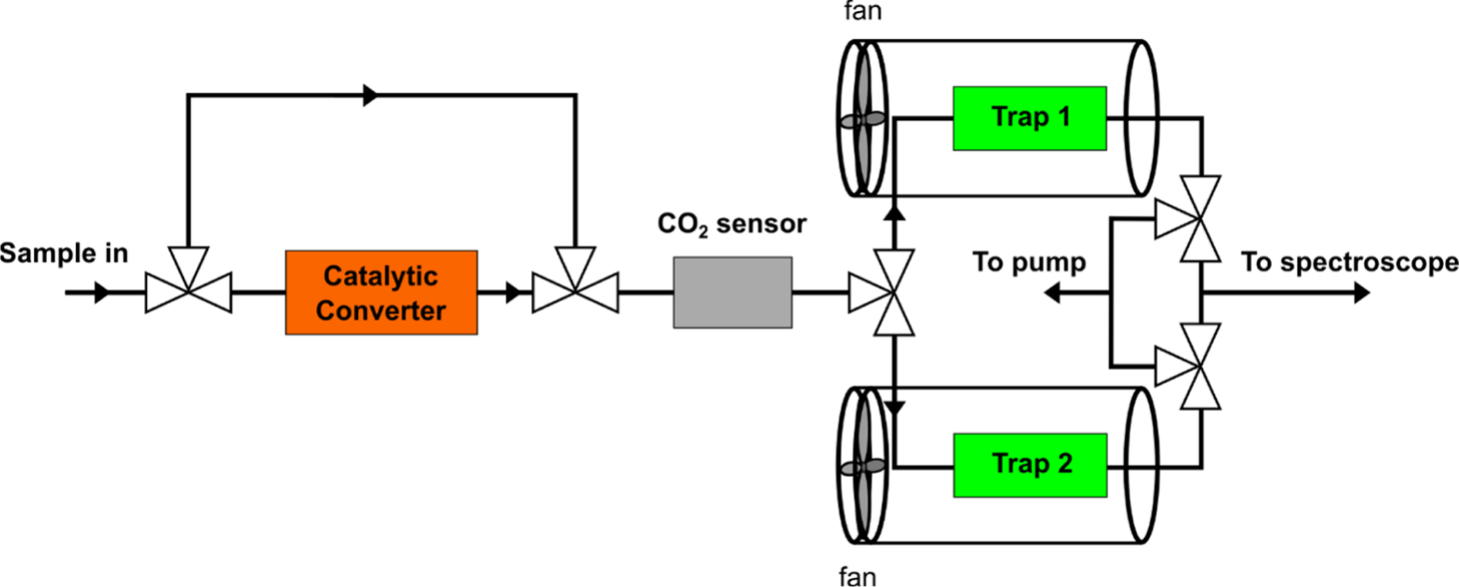
Sample-processing unit. The sample flow direction is controlled by solenoid valves represented by three connected triangles. They enable selecting whether the catalytic converter is used or bypassed and alternating between the two CO_2_ traps.

The radiocarbon concentration is determined by measuring the ^14^CO_2_ concentration with CRDS. The atmospheric CO_2_ concentration is about 400 parts per million (ppm), and similar levels are measured in NPP stacks. To reach the highest sensitivity in radiocarbon detection with CRDS, CO_2_ needs to be first captured and purified from the sample air as the targeted ^14^CO_2_ concentrations are too low to be measured directly at the atmospheric CO_2_ concentration. Therefore, an on-line automated sample-processing unit was coupled to the CRDS instrument. CO_2_ is purified by flowing the sample air through a solid amine-type sorbent ion-exchange resin Lewatit VP OC 1065 from LANXESS. The resin efficiently and selectively adsorbs CO_2_ from air at room temperature, and CO_2_ desorbs by heating the resin to a temperature of 50–100 °C. Two parallel CO_2_ traps were made of aluminum cylinders and filled with the resin. The traps are heated resistively, while active cooling is achieved with heat sinks and fans. The two traps can trap sample air alternately and the sample flow is controlled by solenoid valves as shown in Figure 2. In this configuration, one trap can release trapped CO_2_ to the CRDS unit and cool down, while the other trap collects CO_2_ for the next measurement. A 45 min trapping time was used, after which the CRDS cavity and the trap are connected and pumped to vacuum before releasing CO_2_ by heating the trap. The CO_2_ releasing procedure from the end of the trapping until the C14 measurement starts takes 20 min. The C14 measurement is followed by pumping down the cavity to a 2 mbar pressure for a measurement of CO_2_ concentration in the cavity. In total, the two measurements take 10–15 min, after which the cavity is pumped to vacuum before the next measurement. Meanwhile, the heated trap is flushed with sample air to ensure that it is purged from all the trapped CO_2_. The trap is then actively cooled down back to room temperature with a fan before the next trapping sequence. To capture all the radiocarbon in the form of CO_2_, the sample air is guided before the traps through a palladium catalyst, which was prepared as described in refs (25) and (30). The palladium catalyst converts CH_4_ and possible other hydrocarbons into CO_2_. The measurement of C14 in the form of CO_2_ only is performed by bypassing the catalyst. Downstream the catalyst, a Vaisala GMP343 carbon dioxide sensor is used to obtain the total concentration of the carbon species. In addition, the pressure at the CO_2_ sensor is monitored. After the CO_2_ sensor, the sample flow is guided through a mass flow controller to CO_2_ purification. A diaphragm pump is used to generate the sample flow through the different components of the sample-processing unit.

### Radiocarbon Measurement

To measure the ^14^CO_2_ concentration with CRDS, we targeted the P(20) line at 2209.109 cm^–1^ from the fundamental asymmetric stretching ro-vibrational band ν_3_. The line has been recognized as the most distinct spectral feature of ^14^CO_2_, with the least overlap with other species, particularly with other CO_2_ isotopes.^17,18,23,31,32^ To determine the ^14^CO_2_ concentration, a sum of Voigt profiles is fitted with a non-linear least-square-fitting routine to the experimental data, which was first smoothed with a moving average filter. Other absorption lines of ^12^C^16^O_2_, ^13^C^16^O_2_, ^16^O^13^C^17^O, and ^16^O^13^C^18^O situated in the vicinity of the ^14^CO_2_ line between 2208.9 and 2209.18 cm^–1^ are included in the fitting model.^33−35^ N_2_O lines in the vicinity of the ^14^CO_2_ line were included as well in the fitting model since the trapped sample was not completely pure CO_2_.^35,36^ The amine groups of the resin material degrade slightly when heated, resulting in a trace amount of N_2_O in the released gas.^37^ The measured concentrations of N_2_O after trapping were typically about 2 ppm, which does not interfere with the C14 measurement but must be considered to ensure a good spectral fit. A total of 11 lines were included in the fitting for the ^14^CO_2_ line fit, and they are listed in the Supporting Information. Known N_2_O and CO_2_ line positions were used as anchoring points for the wavenumber calibration, and the scaling was based on an etalon signal measurement. A gas sample with a known ^14^C/C_tot_ ratio of 1.01 ppb prepared earlier in ref (18) was used to calibrate the ^14^CO_2_ P(20) line intensity with the system. The CRDS measurement of the 1.01 ppb calibration sample at a pressure of 10.10 mbar had a ±0.16 ppb uncertainty. The presented uncertainty is derived as relative uncertainty δ*A*/*A*, wherein *A* is the integrated line area of the fit of the absorption line and δ*A* is the uncertainty of the line area derived from the line fit residual. More details about the uncertainty calculation are given in the Supporting Information.

The C14 activity concentration in the collected sample air is calculated using the equation *C*_C14_ = *C*_crds_*C*_air_/*C*_p_ and equation 3 presented in the Supporting Information. *C*_crds_ represents the CRDS-measured ^14^CO_2_ (C14 measurement) concentration, and *C*_p_ represents the CRDS-measured concentration of the purified CO_2_ in the cavity (CO_2_ measurement). The *C*_crds_ and *C*_p_ were determined by *C*_*i*_ = (*A*_*i*_*k*_b_*T*)/(*S*_0*i*_*p*), where *A*_*i*_ is the line area of the targeted absorption line, *S*_0*i*_ is its line strength, *k*_b_ is the Boltzmann constant, *T* is the temperature of the sample, and *p* is the sample pressure. *C*_air_ is the CO_2_ concentration of sample air before trapping, which was measured with the carbon dioxide sensor. The purified CO_2_ concentration was obtained primarily by measuring a ^13^CO_2_ line at 2209.77 cm^–1^. However, in some spectra recorded in the field, the line had shifted to the edge of the recorded spectrum because of temperature drift of the laser control components, and an ^18^O^13^C^16^O line at 2209.81 cm^–1^ was used instead. A total of 14 lines of CO_2_ and N_2_O, which are listed in the Supporting Information, were included in the line fitting for the CO_2_ measurement based on the ^13^CO_2_ line at 2209.77 cm^–1^ or the ^18^O^13^C^16^O line at 2208.81 cm^–1^. The line intensities of the two lines were calibrated with a pure CO_2_ sample with a known isotope composition. For each spectrum of C14 and CO_2_ measurements, 1400 individual ring-down events were recorded, corresponding to 6 min and 2 min acquisition times, respectively. The CO_2_ measurement took only 2 min because the laser emitted a higher power in this wavelength range, resulting in more light coupled to the cavity and a higher acquisition rate. In laboratory tests of the sample-processing unit, a trapping time of 30 min or longer was found sufficient to capture enough CO_2_ for the CRDS measurement, when trapping room air with 430 ppm of CO_2_.

**Figure 3:**
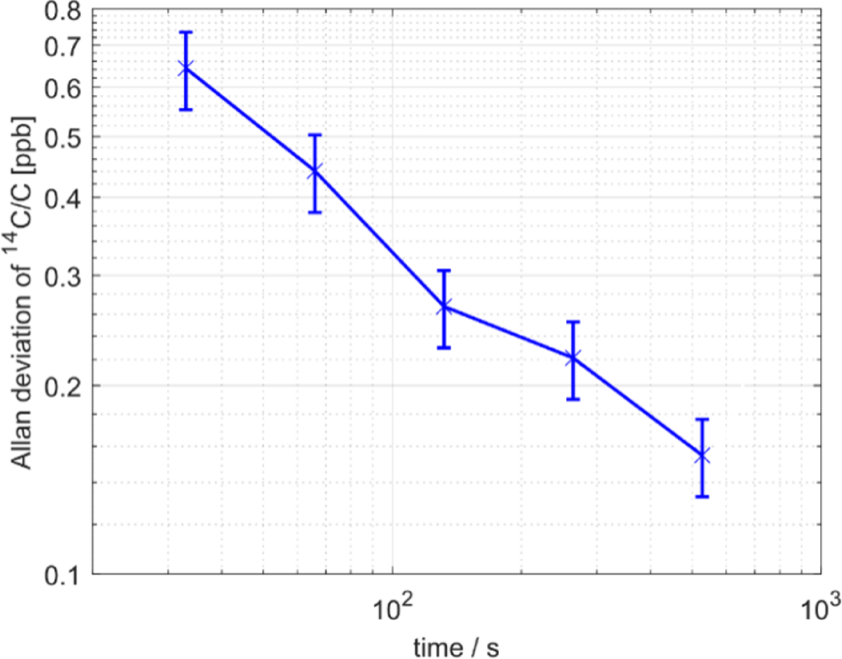
Allan deviation of the C14 measurement. The blue line is the Allan deviation for a ^14^CO_2_ measurement of a 3.6 ppb sample measured at the nuclear power plant. The error bars represent the 1-σ confidence interval.

The precision of the C14 measurement was characterized by performing an Allan deviation analysis, shown in Figure 3. It can be observed that a longer measurement would have increased the precision of the measurement. However, 1400 points per spectrum corresponding to a 6 min acquisition time were selected as compromise between precision and measurement speed. The measurement precision at 6 min was ^14^C/C_tot_ = 0.2 ppb, corresponding to an activity of about 8 Bq/m^3^ in air with a CO_2_ concentration of 400 ppm. The signal to noise ratio of the C14 measurements equalled to 1 for ^14^C/C_tot_ = 0.2 ppb. The detection limit therefore corresponds to the precision determined by the Allan deviation analysis.

The uncertainty in the CRDS measurements mainly originated from electromagnetic noise as all the components of the CRDS setup were situated in close proximity to each other. The largest source of electromagnetic noise was the pump, which could not be completely isolated from the other components in the assembly. When the pump was turned on, it caused an increase from about 11 nanoseconds to 16 nanoseconds in the standard deviation of 1000 sequential ring-down times in an empty cavity. The effect of the increased standard deviation of the ring-down times was accounted for in the uncertainty calculation of the line fitting. The effect of the instrumental noise was greater on the C14 line measurement than on the CO_2_ concentration measurement, for which the absorption lines were stronger. For the total measurement uncertainty, the uncertainties from the absorption spectrum fits for the *C*_crds_ and *C*_p_ values and the uncertainties of the line intensity calibrations were combined using the uncorrelated form of error propagation. The uncertainties of *C*_air_, pressure and temperature were considered negligible compared to the line fitting uncertainties.

### In Situ Measurements

The developed instrument was used to monitor in situ the C14 emissions from the Loviisa nuclear power plant on the south coast of Finland. The measurement campaign took place in the fall of 2019 between September 25th and October 4th, during which continuous automated monitoring of C14 emissions was demonstrated for the first time. Currently, the plant operator measures the radiocarbon content in a radiochemistry laboratory with LSC after collection of the sample to a molecular sieve from the stack gas flow. A few days is the minimum requirement to collect a sufficient amount of CO_2_ for the LSC analysis, and the maximum collection time is typically 2 weeks. The time resolution for this method is poor as the measured radiocarbon activity is the average activity concentration during the collection time: a minimum of a few days. Short time variations are thus undetectable with the current method.

The Loviisa power plant has two pressurized water reactors (PWRs) based on the VVER-440 type, producing 500 MW each. The plant properties regarding radiocarbon emissions are relatively well known.^1,8,38^ In PWR-type NPPs, ^14^CO_2_ is reported to account for 5–26% and the hydrocarbons (mainly ^14^CH_4_ and ^14^C^12^CH_6_) account for 74–95% of the released C14. In Loviisa NPP, an even greater portion of radiocarbon stack emissions has been reported to result from hydrocarbons as only 0.77–10.3% was in the form of ^14^CO_2_ with a 2-month average of 3.8%.^39,40^ In general and in this study, the C14 in carbon monoxide is not differentiated from the measurement of C14 in hydrocarbons because of its minor contribution to the C14 emissions.^8^ In Loviisa NPP, the C14-containing gases are mainly released from the primary water circuit via the off-gas from the primary water degasification and from the outlet water treatment. The off-gas treatment systems, the air ventilated from the plant-controlled area, reactor and auxiliary buildings, and the containment and annulus air are all vented through the stack. Earlier studies at the power plant showed that the off-gas treatment system accounted for 86% of the C14 stack releases.^40^ However, all the pathways of the gaseous radiocarbon within the facility to the stack are not exactly known.

In Loviisa NPP, a fraction of the air exiting through the stack is collected via separate monitoring lines for each reactor to be analyzed for radiocarbon, tritium, iodine, noble gases, and other radioactive contents. The airflow through the monitoring lines is 30–70 L/min, and the residual airflow is returned to the stack exhaust after analysis. The operators collect sample for the conventional radiocarbon analysis with molecular sieves situated on the monitoring lines. For the radiocarbon analysis with CRDS, we connected our sample inlet to the monitoring line before the molecular sieve and returned the residual airflow to the line downstream of the sieve. The airflow during trapping through the sample-processing unit using the catalytic converter was 0.7 L/min for the total radiocarbon analysis and 1.0 L/min for the detection of radiocarbon in carbon dioxide when the converter was bypassed. A 45 min trapping time was used for a single radiocarbon sample in the CRDS measurement to ensure sufficient CO_2_ trapping even when the sample air composition varies. Still, the CRDS measurement was remarkably faster than the few days duration with the conventional molecular sieve–LSC method.

## Results and Discussion

**Figure 4:**
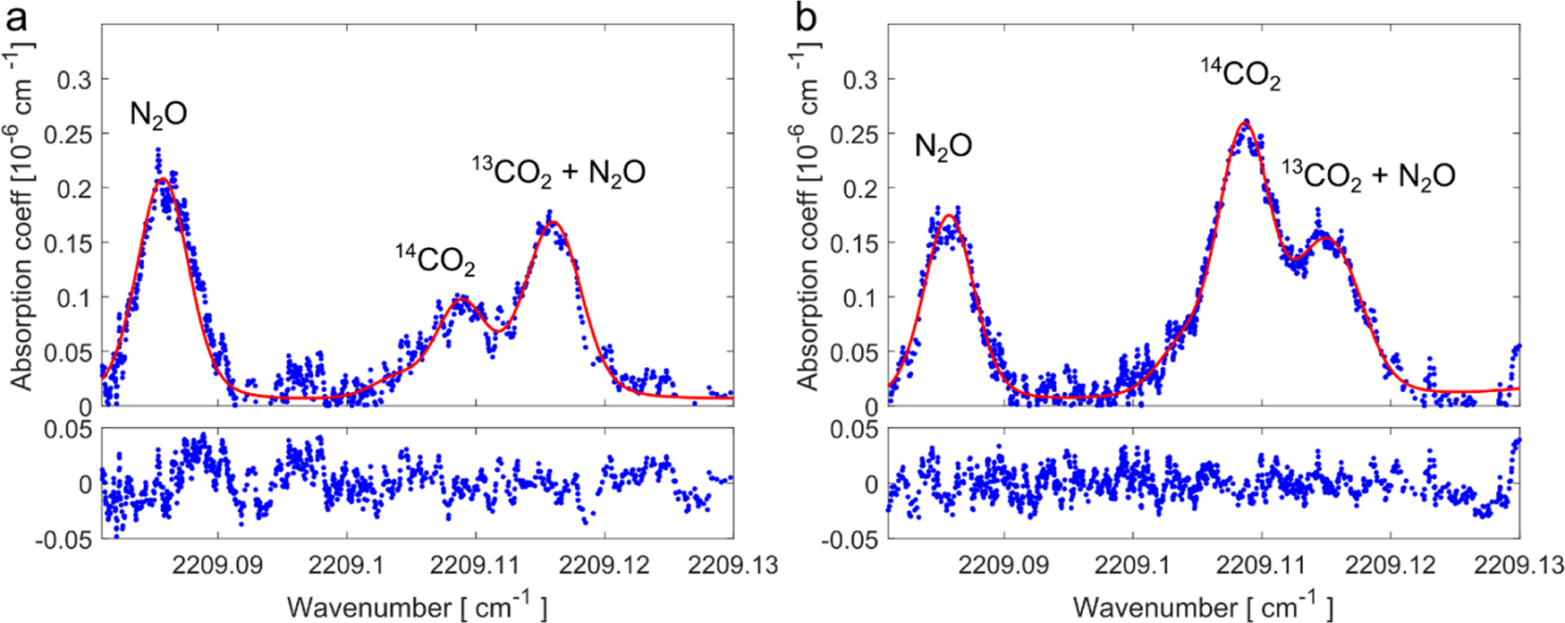
Two absorption spectra recorded from the nuclear power plant stack. The spectra were recorded on September 25th (a) and 26th (b). The ring-down data, shown in blue, are smoothed with a moving average filter with a window size of 10. The red lines represent the fitted sum of Voigt profiles, and the corresponding residuals are shown below. A clear difference in the intensity of the ^14^CO_2_ peak at 2209.109 cm^–1^ is visible. In (a), the mole fraction of C14 was 1.3 ppb, while in (b), it was 4.6 ppb. The N_2_O line at 2209.085 cm^–1^ was used as an anchoring point for the wavenumber scale.

During the field campaign, measurements were performed on 8 different days and 131 unique data points were recorded from the stack monitoring lines. The obtained values are presented in the Supporting Information table. The measurement was fully automated, providing monitoring capabilities day and night. Only minor adjustments were necessary in average once per day. Two example spectra recorded at the plant are shown in Figure 4. A clear difference in the ^14^CO_2_ peak intensities is observed between a measurement from reactor 1 (LO1) on September 25th in (a) and a measurement from reactor 2 (LO2) on September 26th in (b). From the line fitting and the determination of *C*_crds_, *C*_air_, and *C*_p_, the radiocarbon activity concentrations for the two spectra were 52 Bq/m^3^ (^14^C/C_tot_ = 1.3 ± 0.3 ppb) and 189 Bq/m^3^ (^14^C/C_tot_ = 4.6 ± 0.9 ppb) in (a,b), respectively. While the precision of the measurement is determined by the uncertainty of the Allan deviation, the absolute uncertainty is determined by the absolute calibration of the P(20) line intensity using the standardized sample. The absolute uncertainty scales with the mole fractions, causing higher activity values to have a higher total uncertainty. The amount of N_2_O was determined using the line at 2209.085 cm^–1^, and the concentration was 1.6 ppm in (a) and 1.5 ppm in (b). The N_2_O concentrations were typically about 10 times lower than what was reported earlier, when cryogenic cooling was used for CO_2_ purification in combination with catalytic conversion for N_2_O removal.^25^ The two spectra were recorded at a cavity pressure of 7.5 mbar. The purified CO_2_ concentrations in these measurements were 73 and 75% for (a,b), respectively. In addition to CO_2_, the purified gas contains mainly air, which is not completely pumped out of the trap, and water vapor, which co-adsorbs in a small amount to the resin with CO_2_.

The measurements were started on the LO2 stack emissions on September 25th to test the instrumentation in normal operation of the NPP. Eight separate measurement points were obtained with a 45 min CO_2_ trapping time (a graph showing the activity concentration evolution is shown in the Supporting Information). The recorded activity concentrations varied from 42 ± 12 to 68 ± 17 Bq/m^3^ with an average of 56 Bq/m^3^. The instrument was then switched to monitor LO1, which was under maintenance outage, and more variations in the C14 activity concentrations were expected.

**Figure 5:**
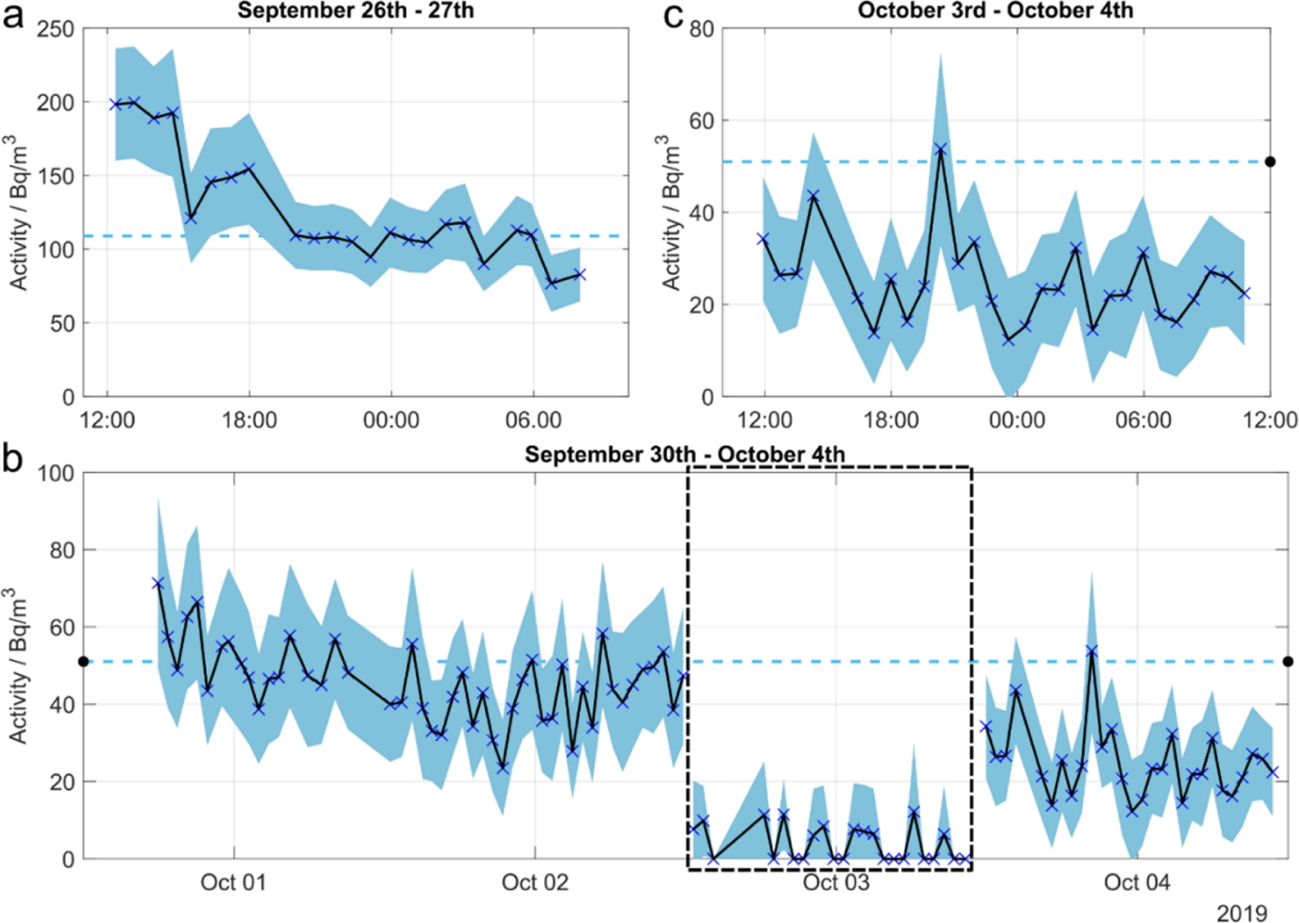
Continuous measurement of radiocarbon activity concentrations over time at Loviisa NPP. The black line shows the evolution of the radiocarbon activity concentration with the blue X’s representing individual data points and the shaded area in light blue the measurement uncertainty. The 1-week C14 activity concentration average measured by the operator with the conventional method (molecular sieve + LSC) is shown with the light-blue dashed line. (a) Radiocarbon activity concentrations of LO1 on the 2 final days of maintenance outage before the reactor startup in the evening of September 27th. (b) 5 days long automated monitoring of LO1 C14 stack emissions. The period inside the black dashed square contains the C14 activity concentrations measured from CO_2_ only without the catalytic conversion of hydrocarbons. (c) Closer visualization on the last ∼24 h of the measurement.

The evolution of the measured radiocarbon activity concentration from LO1 during the measurement campaign is presented in Figure 5. The recorded activity concentrations of a 20 h continuous monitoring from LO1 in the last 2 days of the maintenance outage before the reactor startup are shown in Figure 5a. The first data points obtained from LO1 were significantly higher than those measured earlier from LO2. The activity concentration stayed near 200 Bq/m^3^ for the first 3 h, and the highest activity of the whole field campaign of 199 Bq/m^3^, corresponding to 4.8 ± 0.9 ppb, was measured during this time. Later, the activity dropped to between 75 and 155 Bq/m^3^, resulting in an average activity of 126 Bq/m^3^ for the 2 days. Figure 5a also shows the 1-week average (dashed line at 103 Bq/m^3^) for September 23rd–30th, which was measured by the plant operators with the conventional method. For the earlier week of September 17th–23rd during the maintenance outage, the operator measured a C14 average activity concentration of 261 Bq/m^3^. This comparison demonstrates the added value of this novel method as we can identify the exact time points when the changes between these two values occurred. Conventional methods are not able to capture such fast changes.

A 1-week continuous measurement of the C14 activity concentration in the LO1 stack outgassing is shown in Figure 5b. The average C14 activity concentration during the first 15 h of the measurement was 53 Bq/m^3^, after which a decrease was observed and the daily averages were between 22 and 44 Bq/m^3^ for the remaining days. Figure 5c presents a closer look of the last 24 h of the measurements, where oscillation of C14 activity concentration can be observed between local minima and maxima with about 6 h cycles. The reason for this repeating pattern is not clear since there are multiple compartments containing radiocarbon in the NPP with ventilation cycles that are not synchronized with each other. More data on the nuclear plant systems would be needed to draw definite conclusions for the cause of these fluctuations. The lowest total C14 activity concentration measured during this time was below the detection limit of the CRDS instrument.

During the period highlighted by the black dashed line in Figure 5b, C14 only in the form of CO_2_ was monitored as the catalytic converter in the sample processing unit was bypassed. The measured C14 mole fractions during this period were very close to or below the detection limit of our instrument, with a maximum value of 0.29 ± 0.23 ppb, corresponding to an activity concentration of 11 Bq/m^3^. ^14^CO_2_ therefore accounted for only a small fraction of the total radiocarbon content, with most emissions being in the form of methane and other organic compounds. This is in agreement with the earlier studies made on the PWRs and at the Loviisa NPP, where it was shown that the ^14^CO_2_ concentration varied between only 0.77 and 10.3% of the total radiocarbon, while the rest was in the form of methane and other hydrocarbons.^1,8,38,39^

The average C14 activity concentration measured by the operator over the 1-week period was 51 Bq/m^3^ and is presented by the horizontal dashed line in Figure 5b. The average of the CRDS measurement was 38 Bq/m^3^, although the calculated result is missing data points for about 1/3 of the comparison period. 24 h of the period was used for measuring the radiocarbon only in CO_2_, and the CRDS measurement was started 7 h later than the measurement of the operator. Higher values in the beginning (the previous week average was 109 Bq/m^3^) can explain part of the discrepancy between the averages as well as possible discharge of higher total radiocarbon emissions during the 24 h measurement of radiocarbon in CO_2_ only.

Overall, the C14 activity concentrations measured from LO1 indicate a decreasing trend and leveling after the end of the outage as the reactor operation normalizes. This was also in agreement with the 1-week averages measured by the plant operators. The increased C14 activity concentration during and right after the maintenance outage is likely a result of increased flow through primary water degasification and the off-gas, which is required to degasify the primary coolant before other functions of the maintenance can be performed. The active gas accumulates in the off-gas treatment system and its filters, which delays their discharge to the stacks. The discharge can last for several days after the primary water degasification has ended.

Although the measurements were carried out in an industrial environment, it did not affect substantially the stability of the measurement, thanks to a carefully designed system. The room temperature was varying between 17 and 22 °C, depending on the time of the day. Because the measurement cavity and QCL were temperature-stabilized, they were mostly immune to the temperature changes. The temperature changes had some effect on the overall temperature of the instrument rack including the control electronics, affecting, for example, the wavelength set point of QCL, but these were compensated with minor adjustments once a day.

## Conclusions

The presented CRDS method proved to be very suitable for the monitoring of fugitive radiocarbon emissions and exhibited unprecedented temporal resolution. We have measured fluctuations of the radiocarbon activity concentrations, which could not be captured with the currently available methods. The demonstrated temporal resolution can in the future contribute to a better understanding and control of the dynamics of radionuclide production and transit in NPPs. The CRDS connected with the presented sample-processing unit also allowed the determination of the amount of radiocarbon in different molecular forms. The sample-processing unit can be easily modified to allow simultaneous detection of both the total radiocarbon and the radiocarbon in CO_2_ to provide on-line information about the speciation of radiocarbon emissions. This can give additional information on the processes in the nuclear power plants and, together with the enhanced time resolution, contribute to a better evaluation and control of the radioactive discharges.

The time resolution of the instrument can be improved by reducing the CRDS cavity volume so that less CO_2_ is required and a shorter trapping time is needed. At best, the time interval between measurements can be brought down to few minutes, which can provide even more detailed information on radiocarbon discharges within a nuclear facility. The presented work demonstrates the feasibility of this technique for also measuring other gaseous radionuclides. For instance, tritium is with C14, one of the main components in radioactive gas emissions at nuclear facilities, and can be monitored together with C14 using the same technique.^41^ Demonstrating the applicability of laser spectroscopy for in situ NPP C14 monitoring also highlights the future possibilities of the technique for in situ monitoring of atmospheric radiocarbon. Reaching a sensitivity below the radiocarbon natural abundance of 1.2 ppt can contribute to various other applications as a tool to trace the carbon origin. Such sensitivity has already been achieved in the laboratory^17,19−21^ by other groups. The sensitivity of our CRDS system can, for example, be improved by cooling down the cavity to reduce the interferences from other CO_2_ isotopes by using mirrors with higher reflectivity and a longer cavity to increase the ring-down time or by actively locking the laser to the cavity to increase the coupling and the acquisition rate. More efficient removal of the residual N_2_O will also be needed, as well as a careful characterization of the various isotopic fractionation effects occurring in the sampling system. With these developments, one can envision a complete instrumentation for in situ atmospheric radiocarbon monitoring, which can provide a new means of monitoring the contribution of fossil emissions to global warming. This work is an important step in this direction as it demonstrates optical detection of radiocarbon outside the controlled laboratory environment.

## Supporting Information Available

3D sketch of the CRDS rack assembly, a graph of C14 activity concentrations measured on September 25th from LO2; details of the method; uncertainty calculations of the absorption spectrum line fit; absorption lines for the fitting of the spectrum in the ^14^CO_2_ measurement; and absorption lines used for the fitting of the spectrum in the CO_2_ measurement; activity concentration calculation (PDF)
The measured data points and fit results (XLSX)
