# Supplementary material for: On-Line Monitoring of Radiocarbon Emissions in a Nuclear Facility with Cavity Ring-Down Spectroscopy
Source: Anal Chem. 2021 Nov 24;93(48):16096–104. doi: 10.1021/acs.analchem.1c03814 (PMC8655739; doi:10.1021/acs.analchem.1c03814)
Supplement: Supplementary file 2 — ac1c03814_si_002.pdf [file ac1c03814_si_002.pdf]

Supporting information

On-line monitoring of radiocarbon emissions in a nuclear facility with cavity ring-down spectroscopy

Johannes Lehmuskoski*[a], Hannu Vasama[a], Jussi Hämäläinen[a], Jouni Hokkinen[a], Teemu Kärkelä[a], Katja Heiskanen[a], Matti Reinikainen[a], Satu Rautio[b], Miska Hirvelä[b] and Guillaume Genoud*[a]

[a] *VTT Technical Research Centre of Finland Ltd, P.O. Box 1000, FI-02044 VTT, Finland*

[b] *Fortum Power & Heat Oy, Loviisan Voimalaitos, P.O. Box 23, 07901 Loviisa, Finland*

*Email: johannes.lehmuskoski@vtt.fi

**Email: guillaume.genoud@vtt.fi*

Table of Contents

Figure S1. 3D sketch of the CRDS rack assembly S2

Figure S2. C14 activity concentrations measured on Sept 25th from LO2 ....S3

Uncertainty calculation of absorption spectrum line fit S4

Table S1. Absorption lines used for the fitting of the spectrum in the $^{14}CO_2$ measurement S5

Table S2. Absorption lines used for the fitting of the spectrum in the CO_2 measurement S6

Activity concentration calculation S7

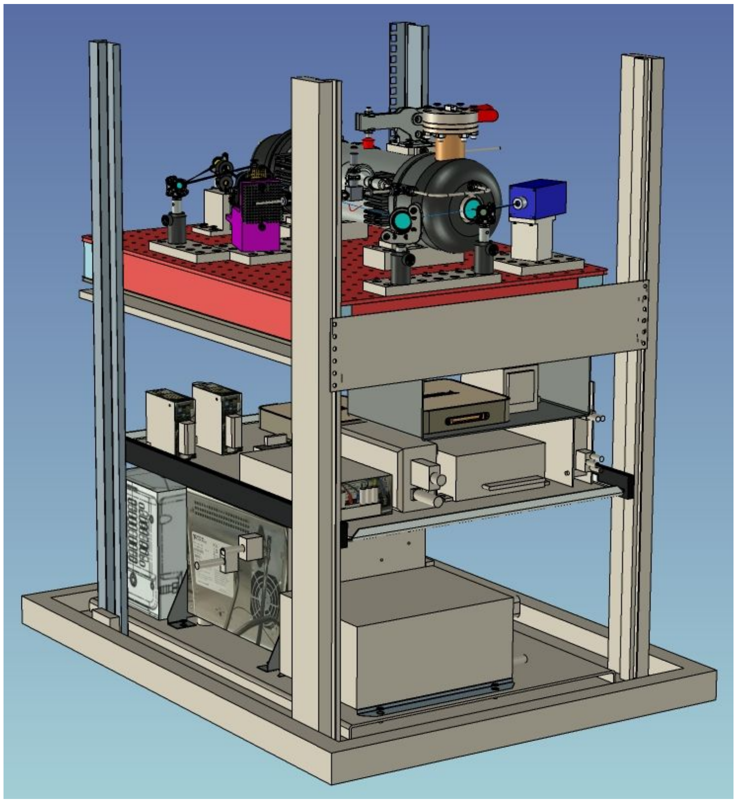

Figure S1. 3D sketch of the CRDS rack assembly. The sketch of the component assembly in the three levels of the transportable instrument rack. The optics are located on the top shelf on a Nexus board. On the second layer are the control electronics and power supplies of the QCL, detector and temperature controllers. On the bottom layer starting from the back are a pump, National Instruments PXI-unit and a piezo control unit.

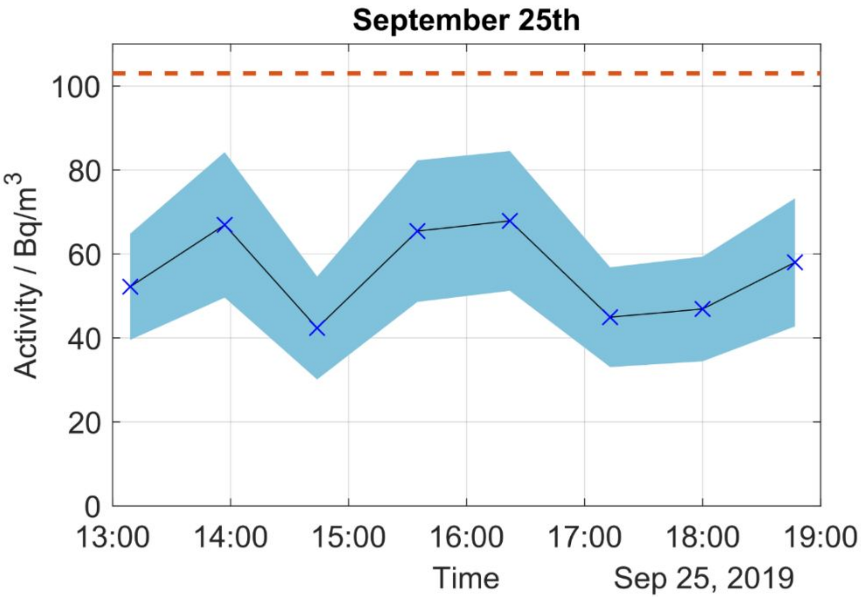

Figure S2. C14 activity concentrations measured on Sept 25th from LO2**.** Blue X's connected with black line represent the eight data points measured from the LO2 on September 25th with the measurement uncertainty shaded in blue. The one-week average activity concentration measured by the NPP operator with the LSC is shown with orange dashed line.

Uncertainty calculation of absorption spectrum line fit.

The uncertainty of the absorption spectrum fit was calculated by determining the error for the area under the Voigt profile fit using the uncorrelated form of error propagation. For simplicity, we calculated the error propagation using a Gaussian profile, as in the low pressure regime the Doppler broadening is predominant:

$$A = \int a\, e^{-\left(\frac{\nu - b}{c}\right)^2} d\nu = ac\sqrt{\pi}, \quad (1)$$

where *A* is the area under the Gaussian curve, *a* is the fit amplitude, *b* is the line centre of the fitted line and *c* is the coefficient related to the Gaussian line width so that the peak full width at half maximum is: $FWHM = 2(ln2)^{1/2}c$. The line area uncertainty using uncorrelated error propagation is then:

$$\delta A = \sqrt{\left(\frac{\partial A}{\partial a}\right)^2 \delta a^2 + \left(\frac{\partial A}{\partial c}\right)^2 \delta c^2} = \sqrt{\pi\left(c^2\, \delta a^2 + a^2\, \delta c^2\right)}, \quad (2)$$

where δa is the amplitude error and δc is the error of the line width coefficient. For the uncertainty calculation of the fitted line area, the standard deviation of the Voigt profile fit residual was used as the amplitude error δa. The laser drift was used as the peak width error δc, which in the instrument was 0.000012 cm^{-1} (0.3 MHz), derived from the QCL current driver noise.

Table S1. Absorption lines used for the fitting of the spectrum in the $^{14}CO_2$ measurement.

Molecule	Wavenumber of the transition line / cm^{-1}	Absorption coefficient / cm^{-1} / (molecule cm^{-2})
$^{13}C^{16}O_2$	2208.946	2.95e-24
$^{14}C^{16}O_2$	2209.109	2.5 e-18
$^{13}C^{16}O_2$	2209.116	4.23e-27
$^{13}C^{16}O_2$	2209.117	1.54e-27
$^{13}C^{16}O_2$	2209.374	9.94e-25
$^{14}N_2{}^{16}O$	2209.037	1.831e-21
$^{14}N_2{}^{16}O$	2209.063	8.628e-22
$^{14}N_2{}^{16}O$	2209.085	3.407e-21
$^{14}N_2{}^{16}O$	2209.103	4.556e-23
$^{14}N_2{}^{16}O$	2209.114	6.612e-22
$^{14}N_2{}^{16}O$	2209.147	9.959e-22

Table S2. Absorption lines used for the fitting of the spectrum in the CO_2 measurement.

Molecule	Wavenumber of the transition line / cm^{-1}	Absorption coefficient / cm^{-1} / (molecule cm^{-2})
$^{16}O^{13}C^{18}O$	2209.711	1.298e-25
$^{16}O^{13}C^{18}O$	2209.747	4.201-27
$^{18}O^{13}C^{17}O$	2209.766	1.951e-28
$^{13}C^{16}O_2$	2209.770	1.56-25
$^{12}C^{16}O_2$	2209.787	4.209e-24
$^{13}C^{16}O_2$	2209.794	2.97e-25
$^{16}O^{13}C^{18}O$	2209.811	1.307e-25
$^{16}O^{13}C^{18}O$	2209.818	4.215e-27
$^{13}C^{16}O_2$	2209.833	1.89e-28
$^{16}O^{13}C^{18}O$	2209.883	3.908e-25
$^{13}C^{16}O_2$	2209.930	3.67e-24
$^{13}C^{16}O_2$	2209.948	7.37e-24
$^{14}N_2{}^{16}O$	2209.747	1.814e-21
$^{14}N_2{}^{16}O$	2209.803	3.367e-21

Activity concentration calculation

The activity concentration was calculated from the C_{C14} using formula:

$$A = \frac{\ln 2}{t_{\frac{1}{2}}} C_{C14} \frac{N_A}{M_{14CO2}} \rho_{CO2}, \quad (3)$$

where $t_{1/2}$ is the half-life of radiocarbon in seconds, N_A is the Avogadro's number, M_{14CO2} is the atomic mass of $^{14}CO_2$ molecule and ρ_{CO2} is the density of carbon dioxide gas.
